# Prototype chemical synapse chip for spatially patterned neurotransmitter stimulation of the retina *ex vivo*

**DOI:** 10.1038/micronano.2017.52

**Published:** 2017-11-06

**Authors:** Corey M. Rountree, Ashwin Raghunathan, John B. Troy, Laxman Saggere

**Affiliations:** 1 Department of Mechanical and Industrial Engineering, University of Illinois at Chicago, Chicago, IL 60607, USA; 2 Department of Biomedical Engineering, Northwestern University, Evanston, IL 60208, USA

**Keywords:** artificial vision, neurotransmitter stimulation, photoreceptor degeneration, retina, synapse chip, visual prosthesis

## Abstract

Biomimetic stimulation of the retina with neurotransmitters, the natural agents of communication at chemical synapses, could be more effective than electrical stimulation for treating blindness from photoreceptor degenerative diseases. Recent studies have demonstrated the feasibility of neurotransmitter stimulation by injecting glutamate, a primary retinal neurotransmitter, into the retina at isolated single sites. Here, we demonstrate spatially patterned multisite stimulation of the retina with glutamate, offering the first experimental evidence for applicability of this strategy for translating visual patterns into afferent neural signals. To accomplish pattern stimulation, we fabricated a special microfluidic device comprising an array of independently addressable microports connected to tiny on-chip glutamate reservoirs via microchannels. The device prefilled with glutamate was interfaced with explanted rat retinas placed over a multielectrode array (MEA) with the retinal ganglion cells (RGC) contacting the electrodes and photoreceptor surface contacting the microports. By independently and simultaneously activating a subset of the microports with modulated pressure pulses, small boluses of glutamate were convectively injected at multiple sites in alphabet patterns over the photoreceptor surface. We found that the glutamate-driven RGC responses recorded through the MEA system were robust and spatially laid out in patterns strongly resembling the injection patterns. The stimulations were also highly localized with spatial resolutions comparable to or better than electrical retinal prostheses. Our findings suggest that surface stimulation of the retina with neurotransmitters in pixelated patterns of visual images is feasible and an artificial chemical synapse chip based on this approach could potentially circumvent the limitations of electrical retinal prostheses.

## Introduction

Visual prostheses are an emerging treatment for certain incurable visual disorders, such as inherited photoreceptor degenerative diseases, with the ultimate goal of restoring vision to the blind through artificial stimulation of neurons. Though many different prosthetic strategies and a variety of stimulation modalities have been investigated, retinal-based prostheses that seek to stimulate the surviving neurons electrically are by far the most clinically successful^1–5^. Electrical-based retinal prostheses, typically interfaced with the macula regions of the retina in either epiretinal (front of the retina) or subretinal (behind retina) configuration, are chips comprising an array of microelectrodes that stimulate retinal neurons electrically, theoretically generating a perception of a point of light at each electrode site. Combined with tiny cameras, these prostheses translate a pixelated image of a patient’s visual environment into electrical pulses delivered to groups of target neurons, predominantly bipolar or retinal ganglion cells (RGCs), through the microelectrode array. By simultaneously activating multiple electrodes, these devices seek to elicit patterned neural responses that closely resemble the real pattern or image and thereby generate specific perceptions in patients^6^.

While many groups worldwide are developing retinal prostheses, the two most successful retinal prostheses that have passed clinical trials and are currently available to patients commercially are the Argus II (Ref. 7) and Alpha-IMS^6^ devices. Clinical studies have shown that these retinal prostheses are generally well tolerated by patients and capable of restoring limited visual pattern recognition, including rudimentary letter reading in patients blinded by photoreceptor degenerative diseases^2,8,9^. However, the vision restored by these prostheses is still well above the legal blindness limit and advancing their capability to further improve the quality of vision has proven difficult due to inherent limitations of electrical stimulation. Specifically, electrical charge density limitations restrict both the size of microelectrodes and the current that can be safely applied to avoid tissue damage^10^. As a result, electrical stimulation of the retina relies on large, widely separated electrodes that stimulate large regions of the retina, which necessarily limits the visual acuities these devices can restore to levels far above the legal blindness limit^6,10^. Second, though electrical stimulation parameters have been optimized to evoke functional RGC responses, electrical current, being an unnatural stimulus, activates all types of retinal cells, including their axons, indiscriminately and creates confusing perceptions in patients^3,10,11^. Therefore, while current electrical prostheses provide some useful vision to patients, there is a definite need for an alternative stimulation strategy capable of restoring high resolution and more naturalistic vision.

One promising alternative proposed to circumvent many of the limitations of electrical-based retinal prostheses is an artificial chemical synapse chip, i.e., a neurotransmitter-based retinal prosthesis that mimics the natural synaptic communication of the retina. An artificial chemical synapse chip would use exogenous neurotransmitter(s) to biomimetically stimulate the surviving retinal neurons of a degenerated retina through the same synaptic pathways used by normal vision. The feasibility of neurotransmitter-based stimulation of the retina in both epiretinal and subretinal configurations has been recently established through *in vitro* studies wherein glutamate, the primary neurotransmitter in the retina, was injected into explanted retinas at isolated single sites through single-port micropipettes^12–14^. While these single-site glutamate stimulation studies have shown that glutamate can be effective and advantageous over electrical current in achieving high spatial resolutions, multisite patterned stimulation of the retina with glutamate, which is critical for translating two-dimensional visual images into afferent neural signals to accomplish complex visual tasks such as navigation and reading, has never been demonstrated before. Multisite patterned stimulation of the retina requires a specialized microfluidic device with multiple independently addressable microports through which stored neurotransmitters can be released selectively and simultaneously. Although two microfabricated devices^15,16^ have been previously developed to demonstrate the technology for an artificial synapse chip, only one of them was a multiport device^16^ and neither device was used to stimulate the retina or retinal neurons. In this paper, we report on the development of a multiport microfluidic device for multisite stimulation of the retina *ex vivo* and the first evidence for spatial pattern stimulation of the retina with glutamate accomplished by interfacing the device with the retina *in vitro*.

## Materials and methods

### Prototype synapse chip device concept and design

**Figure 1:**
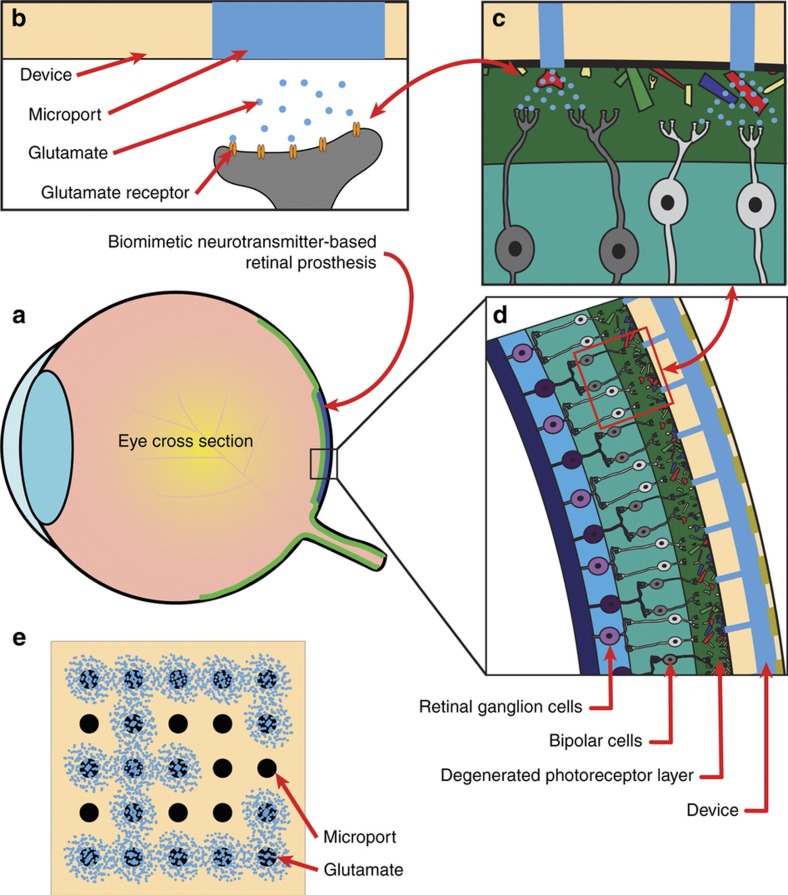
Concept of an artificial chemical synapse chip. (**a**) A cross-section of an eye showing the location in the subretinal space of a degenerated retina where an ideal artificial synapse chip would be implanted (blue shaded region) to stimulate the neurotransmitter receptors of retinal neurons. (**b**) Schematic of an artificial chemical synapse chip device delivering glutamate through a microport targeting the glutamate receptors of surviving retinal neurons at a synaptic junction. (**c** and **d**) Illustrations of an artificial chemical synapse chip device positioned in the former location of the photoreceptor layer with the device microports injecting glutamate in the outer plexiform layer, mimicking the functionality of natural photoreceptors. (**e**) A two dimensional schematic illustrating an example pattern stimulation with an artificial chemical synapse chip. Here, glutamate (blue dots) is injected through a select subset of a 5×5 array of microports of the device to stimulate the retina in a pixelated dot pattern of the letter ‘E.’

Conceptually, an artificial chemical synapse chip is similar to an electrical retinal prosthesis in how it generates a stimulus pattern conforming to a pixelated pattern of a visual image but otherwise differs from all electrical prostheses in two fundamental ways: (1) it would use neurotransmitters to selectively stimulate neurotransmitter receptors instead of electrical fields that stimulate voltage-gated ion channels in the retina, and (2) the interface would comprise an array of independently addressable neurotransmitter delivery ports instead of electrodes. Figures 1a–d illustrates the concept of an artificial chemical synapse chip implanted in the subretinal space of a degenerated retina to stimulate inner nuclear layer (INL) neurons with neurotransmitters released through multiple delivery ports. Figure 1e illustrates an example of how the retina might be stimulated in the pattern of letter ‘E’ by independently activating and injecting neurotransmitters through a subset of the 5×5 array of delivery ports in the device. While an eventual functional artificial chemical synapse chip would be a complex microsystem and include electronic circuits to convert visual image data into appropriate signals to trigger neurotransmitter release through an array of hollow needle-like delivery ports positioned near the INL neurons (as illustrated in Figure 1d), for this initial investigation of pattern stimulation of the retina with glutamate *ex vivo*, we considered a simpler prototype device: a microfluidic device with multiple glutamate reservoirs on one side connected to independently addressable delivery ports on the other side interfaced with *in vitro* preparations of wild-type rat retinas.

**Figure 2:**
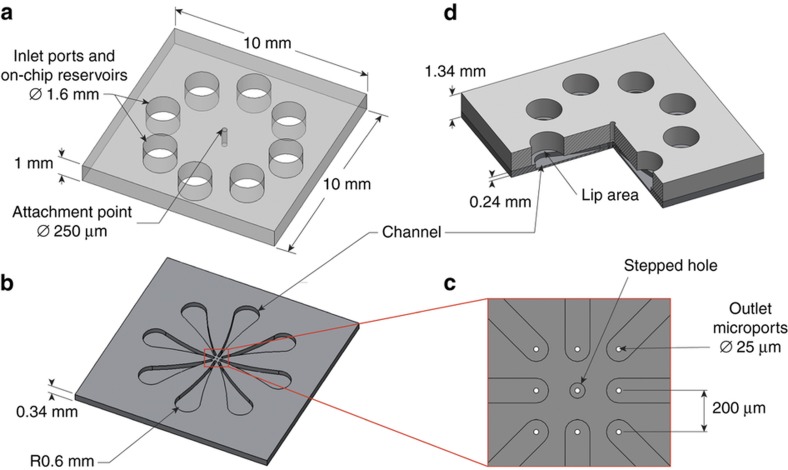
Component design and assembly detail of the prototype artificial chemical synapse chip. (**a**) A solid-model of the top glass layer, 10 mm×10 mm×1 mm in size, featuring eight 1.6 mm-diameter through holes, equally spaced around a circle of 6.4 mm diameter, that serve as on-chip reservoirs and a 250 μm-diameter through hole at the center that serves as an attachment point where the device is anchored to a manipulator arm during experiments. (**b**) A solid-model of the bottom silicon layer, 10 mm×10 mm×340 μm in size, featuring eight 240 μm-deep tapered microchannels with the narrow end of each microchannel feeding into a microport near the center of the chip. (**c**) A close-up view of the eight 25 μm-diameter through holes that serve as microports for glutamate delivery and a 25 μm-diameter stepped hole (25 μm-diameter through hole followed by a 75 μm-diameter and 240 μm-deep hole) at the center that serves as an alignment feature during the device assembly. The eight microports are laid along the periphery of a 3×3 array with a spacing of 200 μm between any two adjacent microports and the larger alignment hole at the center of the chip is not connected to any reservoir and thus not utilized for glutamate injections. (**d**) A quarter cutout-view of the assembled device after integrating the top glass layer and the bottom silicon layer. The hatched region shows the interior details of a reservoir hole aligned with the larger end of a tapered channel with a small lip area at the bottom of the reservoir hole and the smaller end of the tapered channel feeding into a microport. The purpose of the lip area at the bottom of the reservoir is to firmly seat one end of a flexible tube supplying pressure pulses inside the reservoir hole without blocking the channel. This design allows each microport to be addressed independently by actuating the connected reservoir port selectively.

The prototype chemical synapse chip we designed for this *in vitro* study was a multiport microfluidic device tailored for simultaneous pressure injections of glutamate at multiple sites on the outer photoreceptor surface of an explanted retina while sensing the responses of RGCs on the other side of the tissue with a commercial multielectrode array (MEA) system with 60 electrodes. The prototype synapse chip device design was guided by several practical requirements of the experimental setup, viz., the device must: (1) accommodate multiple on-chip reservoirs to store sufficient volume of glutamate to be injected during the experiments, (2) allow millimeter-scale pneumatic connections between the reservoirs on chip and pressure channels of an external multichannel pressure injector for independent actuation of glutamate stored in each of the reservoirs, (3) fit inside the perfusion chamber (internal diameter: 26 mm) of the MEA to interface with the explanted retina placed at the bottom of the chamber without blocking the perfusion flow from the sides and the bottom, (4) have an array of closely spaced independently addressable microports at its bottom to interface with and inject glutamate into the retina, (5) be sufficiently rigid to attach a manipulator arm to position the device so as to precisely locate the glutamate delivery microports over the MEA. To accomplish a prototype satisfying these requirements with simple fabrication processes, a two-layer design with overall dimensions of 1 cm×1 cm×0.134 cm was conceived: a 1 mm-thick top glass layer containing eight 1.6 mm-diameter through holes to serve as reservoirs and a 340 μm-thick bottom silicon layer containing eight 25 μm-diameter microports and eight in-plane microchannels as illustrated in Figures 2a–c. In addition, the bottom silicon and top glass layers featured a 25 μm-diameter stepped hole (25 μm diameter through hole followed by a 75 μm diameter and 240 μm deep hole) and a 250 μm diameter through hole, respectively, at their centers. These central holes were used to align the two layers and attach the device to a manipulator for maneuvering and positioning the device over the retina. The eight microports in the silicon layer were arrayed along the periphery of a 3×3 array with an inter-port spacing of 200 μm, which corresponds to the spacing of the electrodes of the MEA used to record the retinal responses. This layout of the microports permitted the microports to be aligned over the MEA electrodes to inject glutamate into the retina at sites directly above the MEA electrodes and thereby simplify the analyses of spatial spread of glutamate responsive cells relative to the injection sites. The silicon layer also featured eight 240 μm-deep tapered microchannels with rounded ends (Figure 2b). Each microchannel served as a conduit between a reservoir in the top glass layer and a microport in the bottom silicon layer when the two layers were aligned and assembled as illustrated in a cutout view of the assembled device in Figure 2d. The smaller outlet end of each microchannel was shaped to encircle and feed into a microport (Figure 2c) and the larger inlet end of each channel was dimensioned to align the center of the reservoir hole over the center of the rounded end of the channel with a narrow lip under the reservoir hole (Figure 2d). This lip area was provided to firmly seat the end of a flexible tube from a pressure injector source inside the reservoir hole without blocking the channel. This design enabled each microport to be individually addressed by independently actuating the connected reservoir. By selectively actuating a subset of the eight reservoirs with low pressure pulses, pico- to nanoliters of glutamate can be convectively ejected through multiple ports to represent pixelated dot patterns of letters ‘I’, ‘L’, and ‘T.’

### Prototype synapse chip device fabrication

**Figure 3:**
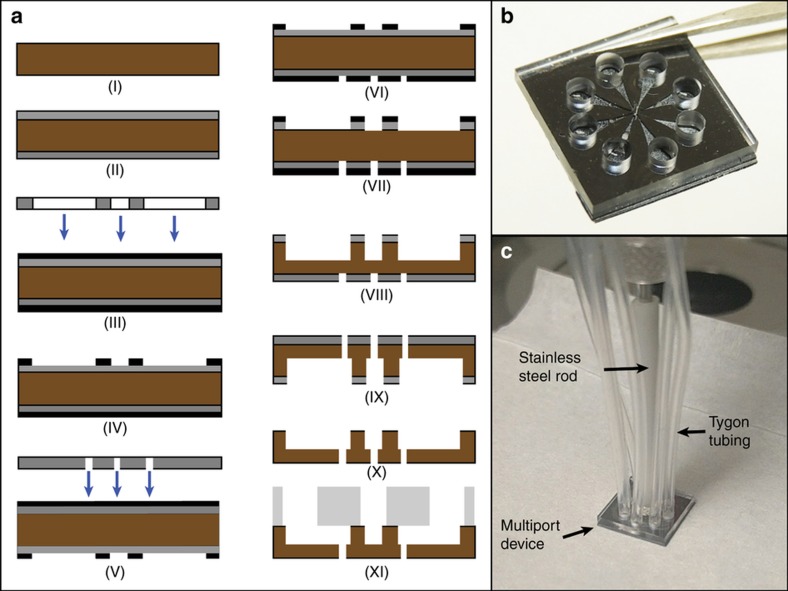
Fabrication and assembly of the prototype artificial chemical synapse chip components. (**a**) Cross-section diagrams illustrating select steps of the process flow for microfabrication of silicon layer. I. Clean silicon wafer, 340 μm thick, II. Deposition of 450 nm-thick aluminum mask layer for the DRIE process, III. Photolithography of microchannels and the larger (75 μm diameter) end of the stepped hole on the top side of the silicon wafer, IV. Development of the exposed regions on the top side with AZ developer, V. Photolithography of microports and the smaller (25 μm diameter) end of the stepped hole on bottom side of the silicon wafer, VI. Development of the exposed regions on the bottom side with AZ developer, VII. Exposed aluminum layer etched with PAN etch, VIII. Tapered microchannels and the larger end of the stepped hole etched to a depth of 240 μm on the top side of the silicon wafer using DRIE, IX. Eight microports and the smaller end of the stepped hole etched to a depth of 100 μm from the bottom side of the silicon wafer using DRIE, X. Aluminum mask layer stripped and the wafer diced into dies, XI. Glass layer chip, fabricated separately, aligned and bonded to the silicon chip using anodic bonding. (**b**) A photograph of the completely fabricated prototype of the device held by tweezers. (**c**) A photograph showing Tygon tubing and manipulation rod attached to the prototype device. Each Tygon tube connects each reservoir in the device to an independent channel of a multichannel pressure injector for actuation and the manipulation rod is used to anchor the device to a manipulator for maneuvering the device during experiments.

The prototype synapse chip device was realized by fabricating the two component layers described above separately and then bonding them together. The top glass layer was fabricated by ultrasonically machining eight 1.6 mm-diameter and one 250 μm-diameter through holes in a 1 mm-thick, double side polished, Schott Borofloat 33 glass at wafer level and then dicing it into 1 cm×1 cm dies (Bullen Ultrasonics, Eaton, OH, USA). The bottom silicon layer was fabricated out of a 340 μm-thick, double side polished, (100) silicon wafer using standard micromachining processes. First, a 450 nm-thick layer of aluminum, which serves as a masking layer for the deep reactive ion etching (DRIE) process, was deposited on a clean silicon wafer using an e-beam vapor deposition process. Then, the eight 25 μm-diameter microports, the 75 μm- and 25 μm-diameter holes for the stepped hole in the center and eight 240 μm-deep tapered microchannels were patterned on either side of the wafer using three masks and double-sided photolithography with backside alignment. These patterned features were then etched using a DRIE process from both sides using aluminum as the masking layer at wafer level. First, the channel side was etched to a depth of 240 μm and then the microport side was etched to a depth of 100 μm so that each microport opens into the smaller end of a tapered channel. After DRIE etching was completed, the aluminum masking layers on both sides were stripped using PAN (phosphoric, acetic, and nitric acid) etch and the wafer was cleaned and diced into 1 cm×1 cm dies. The microfabrication steps for the silicon layer are illustrated in Figure 3a(i–x). Finally, the top glass layer and the bottom silicon layer were aligned at die-level so the reservoir holes are centered above larger inlet ends of the tapered microchannels and the two layers were anodically bonded together at 475 °C and 1100 V. (Figure 3a-xi). Figure 3b shows a picture of the completed device.

Following the fabrication, the device was integrated with peripheral tubing for actuation of glutamate stored in the reservoirs and a miniature steel rod for manipulation of the device. A long flexible tube (Tygon microbore tubing, outer diameter 1.52 mm) was inserted into each of the eight reservoir holes in the glass layer of the device and bonded using water-proof adhesive. The other ends of the tubes were connected to different channels of a multi-channel pressure injector to enable independent actuation of each reservoir. Finally, a miniature stainless steel rod (outer diameter 1.8 mm) was positioned vertically over the central hole on the top glass surface of the device and bonded using water-proof adhesive to enable device manipulation during experiments. Figure 3c shows the flexible tubing and the stainless steel rod attached to the device.

### Prototype synapse chip device characterization

**Figure 4:**
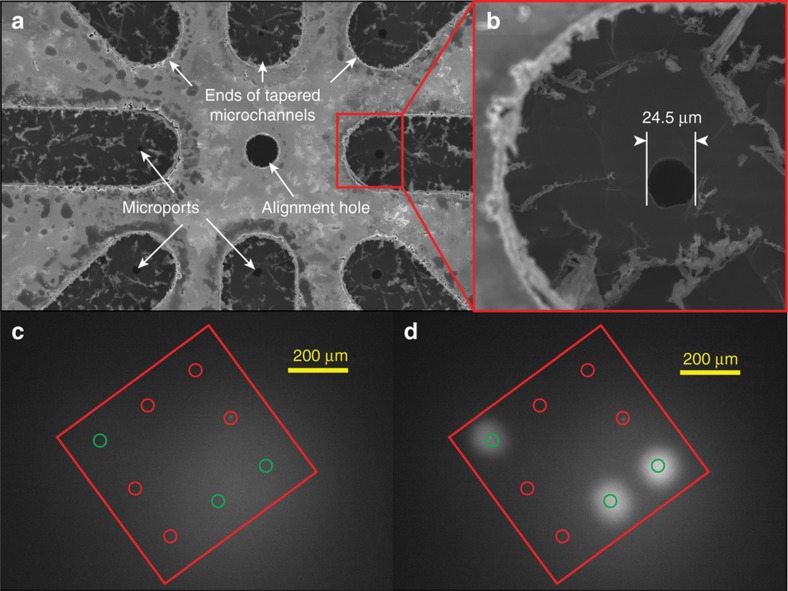
Experimental characterization of the prototype artificial chemical synapse chip device. (**a**) A scanning electron microscope (SEM) image of the eight microports near the center of the silicon layer chip after the DRIE process. The images confirm the openings of all microports and smaller ends of the tapered microchannels feeding into the microports per design. (**b**) An SEM image showing a close up view of a microport hole near the smaller end of the tapered microchannel. The diameters of the holes measured under the microscope from the channel side varied from the intended diameter (25 μm) by about 0.5 μm across all holes yielding an average diameter of 24.5 μm. The variation in hole diameters is attributable to the effects of DRIE etch rate variations within the die including microloading and local pattern density around the microports. (**c** and **d**) Epifluorescent images characterizing the device before and after injecting fluorescein water solution into clear water, respectively. To verify the function and independent actuation of each microchannel and associated microport, water mixed with 0.01% fluorescein dye was injected into a Petri dish containing clear water through all microports, one-by-one, as well as a select subset of microports simultaneously by pneumatically actuating the respective reservoir(s). Active microports (green circles) were identified and distinguished from the inactive microports (red circles) by observing a discernable increase in fluorescence around the active microport(s) immediately after the injections, such as the three microports shown in (**d**), compared to the inactive microports and the initial state of the microports before the injections as in the control image shown in (**c**). This fluorescence microscopy-based characterization of flow through various microports of the device verified that no two channels were inadvertently cross-connected via micro gaps at the interface of the layers due to poor bonding and all active microports could be addressed independently.

Prior to interfacing the prototype synapse chip device with the retina for *in vitro* pattern stimulation, we thoroughly characterized the device geometry and its functionality using both experimental and simulation techniques. First, the features of the etched holes and the channels in the silicon chips post-fabrication (before bonding with glass layers) were inspected under a scanning electron microscope (Figures 4a and b) as well as with a contact profilometer and all etched features were confirmed to conform to the design. The diameters of the microports in the chip used for the prototype device were measured to be in the range 24–25 μm, averaging 24.5 μm on the microchannel side (Figure 4b).

After the component layers were bonded and the device assembled, the prototype was characterized via fluorescence microscopy to confirm fluid flow through each of its reservoir-microport conduits and independent addressability of the microports. For this characterization, the reservoirs of the prototype were connected to different channels of a multichannel pressure injector via flexible Tygon microbore tubing and filled with water mixed with 0.01% fluorescein dye by suctioning the solution through the microports (see Experimental setup section below for more details of the experimental platform). Then, by activating each reservoir independently with pressure pulses, the fluorescein water solution was injected through the microports, one by one, into a Petri dish containing clear water and the functional active microports were confirmed by observing spatially localized increases in fluorescence in the images of the injections (see Figures 4c and d) captured by an epifluorescent imaging system connected to an inverted microscope (Nikon Eclipse Ti-E inverted microscope using Andor Zyla 5.5 sCMOS camera and Nikon Intensilight C-HGFI epi-fluorescence illuminator, Tokyo, Japan).

To estimate the volume of chemical ejected though each microport per injection event, we conducted a finite element analysis of a sectional model of the microfluidic flow through each reservoir-microchannel-microport in COMSOL Multiphysics software. Applying the exact device geometry, material properties and boundary conditions simulating the experimental setup (i.e., 0.69–34 kPa pressure pulses applied at the inlet reservoir port and outlet microports ejecting fluid into water), the model was solved using a time-dependent Navier-Stokes solver. The simulation results were then validated experimentally under similar boundary conditions by a meniscus tracking method (precisely tracking the movement of a meniscus of fluid in the transparent microbore tubing connecting the reservoir and the pressure injector after a finite number of injection events). The simulated and experimental data corresponded well, yielding volumes of approximately 0.5 nl per injection for an input pressure pulse of 3.44 kPa and 30 ms.

### Retinal sample preparation

Retinas were explanted from Hooded Long-Evans rats (PND 25–35, *N*=9, either male or female; Charles River Laboratories, Wilmington, MA, USA) after they were dark-adapted for at least one hour and euthanized by carbon dioxide followed by cervical dislocation. The explanted retinas were placed onto a perforated MEA (pMEA200/30iR-Ti, Multichannel Systems, GmbH) with ganglion cell side towards the electrodes. The MEA chamber was perfused (flow rate 3 mL min^−1^) with Ames medium, which was oxygenated with a medical-grade gas mixture of 95% oxygen and 5% carbon dioxide at room temperature (22 °C). The retinas were left to stabilize from surgical trauma on the MEA perfused with oxygenated Ames medium for at least 30 min before being stimulated visually or chemically. A slight suction was applied at the bottom of the perforated MEA through a perfusion ground plate to prevent the retina from moving due to the perfusion and to maintain a firm contact with the electrodes throughout the entire duration of the experiment. All sample preparation work and stimulation experiments in this study were conducted under dim red illumination to preserve the light sensitivity of photoreceptors during data collection and all recordings were conducted at room temperature.

All animal experiments were conducted in accordance with the guidelines outlined by the National Research Council's Guide for the Care and Use of Laboratory Animals. All experimental protocols were reviewed and approved by the Institutional Animal Care and Use Committee of the University of Illinois at Chicago.

### Experiments

#### Experimental setup

**Figure 5:**
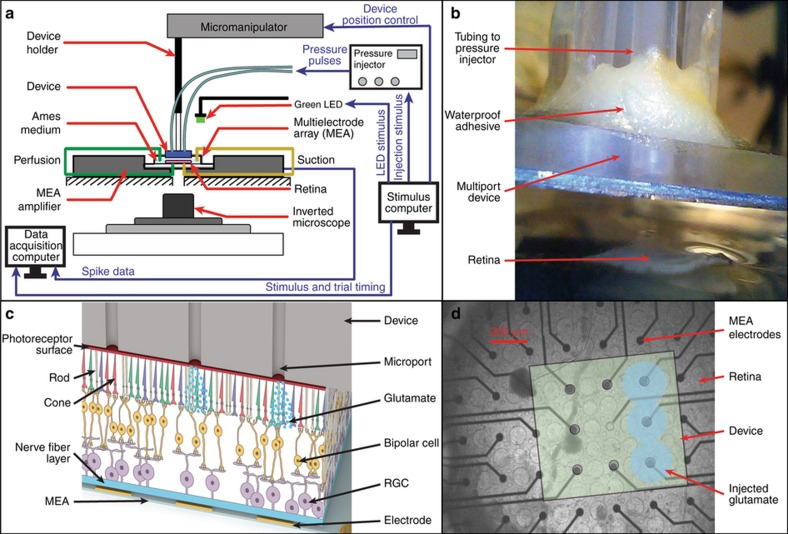
Experimental setup and close-up details of the device interfaced with the retina. (**a**) Schematic of the experimental platform showing the arrangement of various components. Retinas extracted from wild-type rats were placed onto a perforated multielectrode array (pMEA) with the retinal ganglion cells (RGCs) in contact with the electrodes atop an inverted microscope to enable visualization of the retina and pMEA. To keep the retina healthy, it was perfused with Ames medium from both the top and bottom of the pMEA. A green LED controlled by signal from a dedicated computer was used to visually stimulate retinal neurons before the device was interfaced. The synapse chip device filled with glutamate was maneuvered and interfaced with the retina by means of a manipulator arm controlled by the computer. Simultaneous multisite injections of glutamate through multiple microports of the synapse chip device were accomplished by pneumatically actuating the microports using a multichannel pressure injector system (also controlled by the stimulus computer). Retinal response data were acquired from the MEA electrodes into another dedicated data acquisition computer. The LED and pressure injector trigger signals from the stimulus computer were also acquired into the data acquisition computer to synchronize the timings of the stimulation events and neural responses recorded for later data analysis. The flow of output signals and input data throughout the setup is represented by the blue lines with labels above. (**b**) A close-up view of the synapse chip device being lowered and aligned with the MEA electrodes just prior to its interfacing with the retina, which was visually confirmed by observations through the inverted microscope. (**c**) A cross-section diagram of the synapse chip device interfaced with the retina and glutamate being injected into the retina through a select set of microports aligned over the MEA electrodes. The diagram shows schematically the anatomy of a wild-type retina interfaced with the device microports at the top surface and the MEA electrodes at the bottom surface as well as the various interconnections between different retinal neuronal layers including the photoreceptors (top layer), bipolar cells (middle layer), and RGCs (bottom layer). (**d**) An overlay picture of the device microports aligned with the electrodes of the MEA as observed and imaged through the translucent retina tissue under an inverted microscope. This alignment allows the glutamate injection sites to be precisely referenced to the centers of the MEA electrodes, which facilitates the analyses of spatial spread of glutamate injections (blue shaded circles) as well as glutamate responsive cells relative to the injection sites. The picture depicts an example where injections of glutamate (three blue shaded circles) represent a dot pattern of the letter ‘I’ character by simultaneously activating three microports along the right edge of the device.

A special experimental platform (schematically illustrated in Figure 5a) was built to enable glutamate injections from the prototype synapse chip device into retinal tissue from the photoreceptor (top) side while simultaneously recording the responses of retinal neurons at multiple sites on the other (bottom) side contacting the electrodes of the perforated MEA. The 60 electrodes of the perforated MEA, each 30 μm in diameter, were laid out in a grid (8×8) pattern with an inter-electrode spacing of 200 μm. The extracellular voltages of activated retinal neurons picked up by all 60 electrodes of the perforated MEA were amplified and acquired by a computer through an MEA system with MC-Rack software (MEA1060, Multichannel Systems, GmbH). The MEA amplifier system along with the perfusion ground plate was placed over an inverted optical microscope (Nikon Eclipse Ti-E) to observe the retina and interface the device with the retina following visual stimulation. To assess the health of the retina before interfacing the prototype synapse chip device and to permit us to compare glutamate evoked responses with light evoked responses, a green (570 nm) light-emitting diode (LED) was used to visually stimulate the retina with full-field flashes of light from the top.

The prototype synapse chip device was held in a three-axis motorized, precision manipulator (MP-285, Sutter Instruments, Novato, CA, USA) by means of the miniature stainless steel rod and maneuvered to a position over the MEA. The initial location of the device as held by the manipulator arm and the locations of the MEA electrodes were spatially mapped and referenced to a common zero reference point. Next, the ends of the flexible tubing from the eight reservoirs were connected to eight independent channels of an 8-channel pressure injector system (Pressure Injection System PM-8, Harvard Apparatus, Holliston, MA, USA). Lastly, glutamate solution (1 mM; Sigma-Aldrich, St Louis, MO, USA) was prepared in Ames Medium (Sigma-Aldrich) and approximately 6 μL of the glutamate was loaded into each of the eight reservoirs of the device by individually suctioning stock glutamate solution from a Petri dish until each reservoir had been filled. To achieve repeatable, high accuracy spatiotemporal modulation and synchronization of the glutamate and visual stimulus events and precise positioning of the device over the MEA electrodes in each experiment, all of the main instruments (pressure injector, LED light source, and the manipulator) in the setup were computer controlled via a digital-to-analog DAQ board (PCI-6251, 16-bit, National Instruments) driven by custom scripts coded in LabView (National Instruments, Austin, TX, USA).

#### Prototype synapse chip device interface with the retina and glutamate injections

Before interfacing the prototype synapse chip device with the retina and starting the glutamate stimulation protocol, the health of the retina following surgery was assessed by observing the spontaneous and light-evoked responses of RGCs for about 30 min. The spiking RGC units that exhibited robust light responses and the MEA electrodes that registered these visual responses were recorded. Then, the prototype synapse chip device held by the manipulator arm was moved over the area of the MEA electrodes that registered robust visual responses and aligned to precisely position the outlet microports of the device vertically above the target electrodes. Once aligned over the target electrodes, the device was lowered until the microport side of the device was in contact with the retinal surface, which was visually confirmed by observation through the inverted microscope. Figure 5b shows a close-up view of the device positioned over the retina prior to the interfacing and Figure 5c shows a schematic of the cross-section of the interface with the delivery ports of the device aligned with the electrodes of the MEA.

After the device was interfaced with the retina, a subset of the eight reservoirs on the device were simultaneously activated using a pulsatile pressure (0.69–34 kPa and 30 ms pulses) triggered by the corresponding channels of the pressure injector, thereby causing small boluses of glutamate stored in those reservoirs to be convectively injected at the retina surface through the microports. The microports through which glutamate was injected combine to represent a unique pixelated dot pattern of an alphabet character, like the way tiny printed dots combine to form a letter in an inkjet printer. For example, Figure 5d shows an overlay image of the device and the MEA electrodes where a set of three active microports along one edge of the device through which glutamate was injected (three blue shaded circles) combined to form a dot pattern of a character resembling an ‘I’. By similarly activating a different set of microports with identical pressures and pulse times simultaneously, patterned injections in other pixelated character forms such as ‘L’ and ‘T” were accomplished. Each pattern stimulation experiment consisted of a series of 30 trials of injections with an interpulse duration of 3 s, and for all injection trials, the RGC spike responses to the patterned glutamate injections at the surrounding electrodes were recorded.

#### Neural response data acquisition and analysis

**Figure 6:**
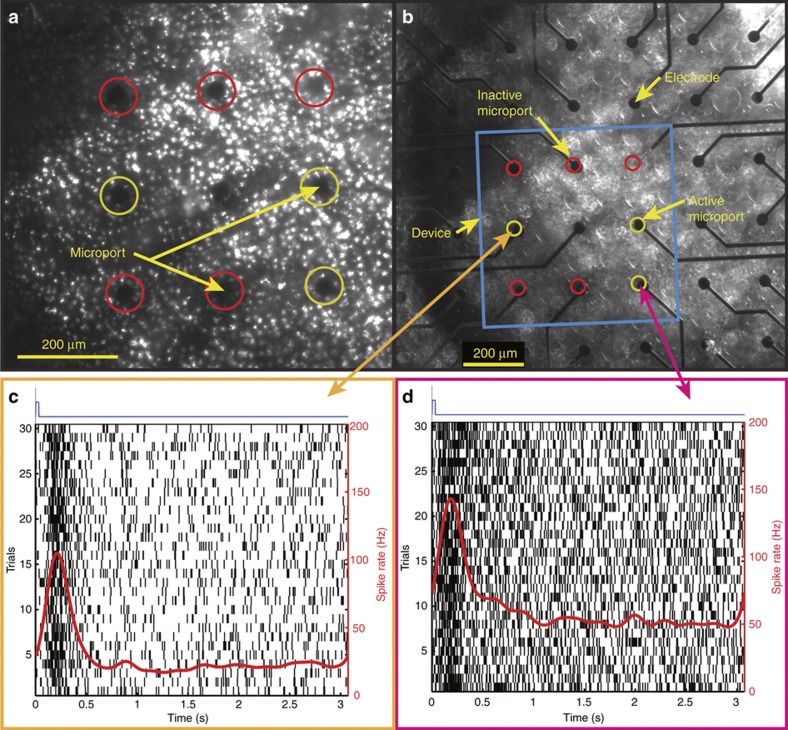
Synchronized multisite glutamate injections through multiple microports of the device simultaneously evoke robust responses from RGCs near the injection sites. (**a** and **b**) Brightfield microscope images of the synapse chip device interfaced with the retina as viewed under an inverted microscope from below through the translucent retinal tissue, with the motorized vertical *z* axis focus locked to the bottom surface of the device depicting the microports and the top surface of the MEA depicting the electrodes, respectively, during a multiport patterned stimulation of a sideways and inverted ‘L’ character. In both pictures, the overlaid red and yellow circles highlight the locations of the inactive and active microports, respectively. (**c** and **d**) Representative examples of two RGC responses that were detected on electrodes directly under the injection sites at the two ends of the ‘L’ character, indicated by the orange and magenta arrows, respectively. Each plot shows the representative spike rate and raster response from a unique RGC under the injection site where the vertical black lines display the timing of individual spikes and are arranged vertically by trial. The red lines show the Gaussian smoothed spike rate for each cell in response to glutamate injections (blue traces at the top display the time-course of glutamate injection). As can be seen, glutamate evoked robust transient excitation of both cells with small time widths comparable to those evoked by visual stimulation but significantly faster response latencies.

Spikes of all RGCs responding to glutamate injections through active microports of the device were acquired from all electrodes in the immediate vicinity of the injection sites. Figure 6a shows a brightfield image of the device microports as viewed from below through the translucent retina under the inverted microscope (Nikon Eclipse Ti-E equipped with Perfect Focus System) with its motorized vertical *z*-axis focus locked to the interface between the device and the retina for the case when glutamate is injected through three microports (highlighted by yellow circles) forming a dot pattern of a character resembling a sideways and inverted ‘L’. Figure 6b shows a brightfield image for the same case with the microscope’s vertical *z*-axis focus locked to the interface between the retina and the MEA electrodes, revealing the MEA electrodes under the active microports from which the RGC spikes were recorded. Figures 6c and d show plots of representative spike rate and raster responses of two RGCs recorded from two electrodes directly under the injection sites defining the two ends of the ‘L’ character. The vertical black lines and the red contoured lines in the plots display the timing of individual spikes for various trials and the Gaussian smoothed spike rates, respectively, for these cells in response to glutamate injections whose time-courses are indicated by blue traces at the top of the plots.

RGC spikes recorded with the MEA system using MC_Rack software were acquired at 10 kHz sampling rate with a high-pass filter (200 Hz cutoff) and an amplitude threshold of approximately −16 μV. Spikes were sorted using a MATLAB wavelet clustering package, Wave_clus, developed by the Quiroga *et al.*^17^. Following spike sorting, unit responses were analyzed with custom MATLAB code to produce peristimulus time histograms (PSTHs), such as the ones shown in Figures 6c and d, using Gaussian kernel density estimation to average spike rates across trials^18^. PSTHs were further analyzed by extracting the temporal characteristics of spike rate responses including the time width and response latency. The time width of responses was calculated as the width at half maximum amplitude while the response latency was determined to be the first time point of the width^14^. These analyses were performed on RGC response data corresponding to both chemical and visual stimulation to facilitate comparisons of the spike rate temporal characteristics. The response latency and time width of RGC responses to visual and multisite chemical stimulation were statistically compared in matched pairs using non-parametric two-sided Wilcoxon signed-rank tests because the data were non-normally distributed. Responsive units were identified using a fano factor derived response variable (for details see Rountree *et al.*^12^) and further filtered by excluding units with spike rates less than 3 Hz to eliminate signals uncharacteristic of typical neural responses.

We used the above neural response data to characterize two important functional traits of an artificial chemical synapse chip: (1) its ability to drive RGC responses that closely follow intended glutamate injection patterns, and (2) its ability to localize the responses around each injection site, potentially permitting high visual acuity. Both the patterned responses of RGCs and spatial localization of glutamate stimulation were investigated by mapping the locations of glutamate-responsive RGCs exhibiting somal spike shapes using the vectors separating electrodes recording responsive units from the glutamate injection sites. Units exhibiting somal, as opposed to axonal, spike shapes were specifically utilized since they are more closely colocalized with glutamate receptors (for details see Inayat *et al.*^14^), the intended primary targets of this neurotransmitter stimulation. For each stimulation pattern, these vectors were assembled into a 2D histogram via spatial binning to represent the approximate locations of electrodes where responsive RGCs were recorded. To quantify the spatial localization of glutamate stimulation for individual microports, the radial distances separating responsive units from the microport of interest were extracted from the corresponding 2D spatial spread data and used to determine how responsive cells were spatially distributed. The spatial distributions for individual microports were then averaged to estimate the spatial distribution of a typical microport.

## Results

### Multisite stimulation elicited patterned RGC responses

**Figure 7:**
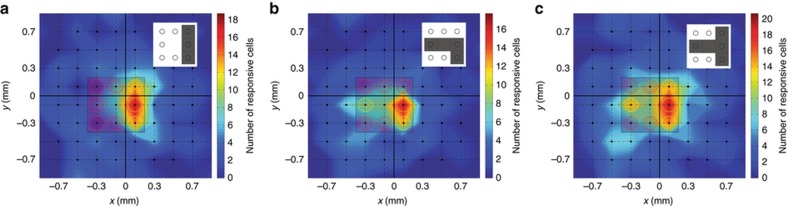
Multisite patterned glutamate injections through the prototype synapse chip device elicit RGC responses in patterns resembling the injection patterns. (**a**–**c**) Two dimensional color histogram plots depicting the spread of somal RGC responses (warmer colors representing larger numbers of responsive cells) to glutamate injections through a select set of microports simultaneously representing pixelated dot patterns of characters ‘I’, sideways and inverted ‘L’ and sideways ‘T’, respectively. In each plot, the inset at the upper right corner shows the corresponding glutamate injection pattern (forms represented by small gray squares) and the responses are overlaid with the device microports (active microports represented by yellow circles and inactive microports represented by red circles) and the 60 electrodes of the MEA (the black dots at the intersections of the 8×8 dashed grid lines, with the exception of the four corner intersections, represent the locations of the 60 electrodes). By visual inspection of the RGC response plots and consideration of patterns formed by warmer colors in each plot, RGC response patterns with high correspondence to the glutamate injection patterns can be readily discerned, thus confirming successful patterned stimulation of the retina with glutamate.

To investigate patterned stimulation with glutamate, we conducted a total of 89 sets of multisite stimulation experiments by interfacing the prototype synapse chip device with 9 retinas (*N*=343 responsive RGCs, including retinas and RGCs used for spatial localization studies discussed in the following section) and injecting small boluses of glutamate through different sets of microports forming dot patterns of three distinct characters: ‘I’, sideways ‘L’ inverted and sideways ‘T’. Each multisite injection pattern elicited RGC spike rate responses, such as the representative responses for two RGCs shown in Figures 6c and d, that were transient in nature with significantly faster response latencies (median 150 ms; *P*<0.001) and insignificantly smaller time widths (median 280 ms; *P*=0.15) compared to light evoked responses. We determined the patterns formed by these RGC responses by summing the spatial distributions of RGC responses to each pattern across all 9 retinas that were stimulated and produced a cumulative map of the responses for each type of pattern. Figure 7 shows 2D histogram plots of the spatial distributions of RGC responses, overlaid with the device microports, to three distinct injection patterns: ‘I’ (Figure 7a), sideways and inverted ‘L’ (Figure 7b) and sideways ‘T’ (Figure 7c). The inset near the upper right corner of each panel of Figure 7 shows the injection pattern (forms represented by small gray squares) and the color spread in each plot indicates the number of glutamate responsive cells at any given location as quantified by the color bar on the right with warmer colors representing higher densities of responsive RGCs. Each histogram plot displays warm colors under each active microport, indicating successful stimulation of a large proportion of RGCs under these injection sites. A particularly higher concentration of warmer (redder) colors, indicative of a greater than average number of glutamate-responsive RGCs, is noticeable under the active microport located in the middle of the three microports along the right edge of the device. This apparent higher density of responsive RGCs is likely due to overlap of glutamate spreading from adjacent injection sites, around this microport, an explanation consistent with the measured median spreads for glutamate as discussed in the following sections. Contrastingly, the noticeable sparseness of responsive RGCs at the center for injection patterns representing sideways and inverted ‘L’ and sideways ‘T’ (Figures 7b and c) is due to the lack of a microport to inject glutamate at the center of the device. Nevertheless, in all three plots (Figures 7a–c), RGC response patterns clearly approximate the respective injection patterns.

### Multisite glutamate stimulations are spatially localized

**Figure 8:**
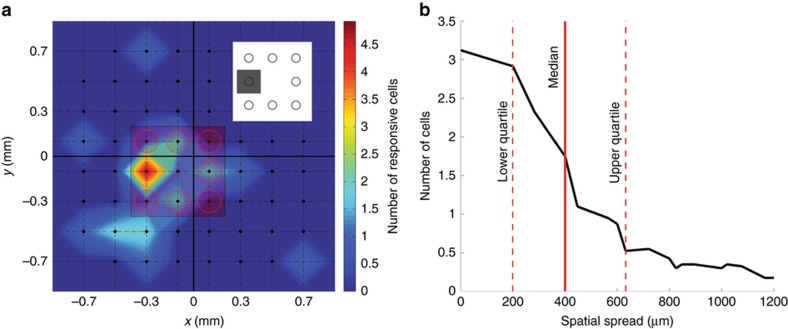
RGC responses to glutamate injected through any single-microport of the multiport synapse chip device are spatially localized. (**a**) A color map displaying the spatial spread of responsive somal RGCs (warmer colors representing larger numbers of responsive cells) to glutamate injected through a representative microport on the left edge of the device (indicated by the small gray square in the inset shown at the upper right corner). The RGC response plot is overlaid with the locations of the device microports (the single active microport represented by a yellow circle and all other inactive microports represented by red circles) and the 60 electrodes of the MEA (the black circles at the intersections of the 8×8 dashed grid lines, with the exception of the four corner intersections, represent the locations of the 60 electrodes). As can be clearly discerned via visual inspection of the plot, RGC responses to glutamate injected through this representative microport were spatially localized with the highest density of responsive cells located near the center of the MEA electrode located directly under this injection site. The median spread of the glutamate-responsive RGCs under this injection site was found to be 400 μm with lower and upper quartiles of 200 and 630 μm, respectively. (**b**) The average spatial distribution of RGCs responding to glutamate injections through individual microports plotted as a function of the radial distances of the RGCs from the center of the electrode under a typical injection site. The plot shows that a relatively high proportion of glutamate-responsive cells were located at or very near the electrode under the typical injection site but a small number of these glutamate-responsive cells were located as far as 1200 μm (which is within the farthest span of ~1720 μm between the corner electrodes of the MEA) from the same electrode. The solid and the dashed red vertical lines indicate the median and the lower/upper quartiles of the spread of the glutamate-responsive cells, respectively.

To investigate the spatial localization of glutamate stimulation with our prototype synapse chip device, we examined the spread of retinal responses to single-microport injections with each retinal sample (*N*=9 as mentioned above). Figure 8a shows the 2D spatial spread of RGCs responding to injections at a representative active microport (indicated by the yellow circle) where warmer colors represent higher numbers of glutamate-responsive cells. Although some glutamate-responsive cells were found far from the microport, the highest concentration of responsive cells were found to be near the center of this microport, indicating a high spatial localization of glutamate stimulation. To ascertain this trend more quantitatively across all microports, we further analyzed the 2D spatial spread data corresponding to all active microports and determined the spatial distributions of glutamate-responsive RGCs in terms of their radial distances from respective injection sites. We found that the average spatial distribution of glutamate-responsive cells (i.e., the number of responsive cells at any radial distance from an injection site averaged across all active microports), which is plotted in Figure 8b, was spatially localized with a median spread of 400 μm and lower and upper quartiles of 200 and 630 μm, respectively.

## Discussion

Electrical-based retinal prostheses have emerged as the most viable and promising strategy to restore vision in blind patients affected by photoreceptor degenerative diseases over the last two decades. Electrical stimulation of neurons, which as an approach dates back to the 18th century^19^, seeks to elicit neural responses by means of electrical charges applied through an array of microelectrodes interfaced with the retina. Significant progress has been made in restoring useful vision through electrical stimulation with retinal implant devices over the last two decades and two devices have received market approval for treating patients with hereditary retinal degenerative diseases^6^. Yet, this technology is unable to restore high-resolution naturalistic vision due to the characteristics of the electrical current distribution through a large array of electrodes. Two fundamental limitations of electrical stimulation are its inability to: (1) selectively stimulate various retinal neurons as the natural retinal circuitry does, and (2) enable high resolution distributed stimulation since one needs a large array of tiny electrodes to cover the fine retinal cellular mosaic^10^. Therefore, it is worthwhile to explore alternative strategies and agents that could stimulate the retina more effectively by engaging visual pathways biomimetically.

The work presented in this paper is motivated by the opportunity to enable a biomimetic stimulation strategy as a better alternative to electrical-based retinal prostheses: an artificial chemical synapse chip that closely mimics the functional characteristics of the natural photoreceptors lost to degenerative diseases and, ideally, takes over their functionality when implanted in the subretinal space, replacing the degenerated photoreceptors. Conceptually, an artificial synapse chip would comprise a large array of tiny independent reservoirs that store neurotransmitters and release them on to the synaptic junctions of the surviving INL layer through nanoports in response to light, similar to how the photoreceptor layer works. By dispensing neurotransmitters in patterns that approximate the visual image, an artificial chemical synapse chip could potentially stimulate the retina to produce more naturalistic and high-resolution vision perception in patients implanted with such a device than can an electrical prosthesis. While high-resolution, single site neurotransmitter stimulation of the retina has previously been demonstrated using micropipettes^12^, this work is the very first attempt towards pattern stimulation of the retina with neurotransmitters by interfacing a multiport microfluidic device, a basic prototype of an artificial synapse chip, with the retina *ex vivo*. Although an artificial chemical synapse chip as an implant is intended for ultimately interfacing with photoreceptor degenerated retinas, we intentionally chose wild-type retinas to study pattern stimulation with glutamate in this initial investigation in order to permit quick assessment of the functional health of the retina *ex vivo* with visual stimulation before glutamate stimulation experiments lasting over several hours. Thus, the dual goals of this initial work were to: (1) fabricate a custom microfluidic device to demonstrate the concept of a chemical synapse chip and (2) using the prototype synapse chip device, investigate pattern stimulation of the wild-type retina, i.e., if glutamate injected simultaneously at multiple sites in pixelated dot patterns of alphabet characters would focally stimulate the RGCs and evoke responses in similar patterns.

Three important features of an ultimate chemical synapse chip that impact the spatiotemporal resolution and spatial localization of pattern stimulation are the number (array density) and size of the neurotransmitter delivery ports and the actuation frequency of glutamate injections. For this initial study, we designed and fabricated a multiport microfluidic device with only eight microports since only eight independent channels were available in the pressure injector used to actuate each port independently. Even with a total of just eight injection microports, we were able to inject glutamate in distinct alphabet character patterns by simultaneously activating only—three to four microports at a time. The analyses of RGC responses to various patterned glutamate stimulations revealed that the glutamate-driven response patterns closely resemble the injection patterns, indicating the possibility of translating a pixelated pattern of a complex visual scene into more naturalistic afferent retinal signals via exogenous neurotransmitters. Each microport in the prototype device was sized to be on the large side (25 μm diameter and 100 μm deep) mainly to prevent clogging of the ports due to perfusion currents in the MEA chamber during interfacing with the tissue *in vitro* and stimulation experiments. Even with such relatively large microports in this rudimentary device, the stimulations were focal and the median spread of the neural responses were contained within 400 μm, indicating a high spatial resolution. The spatial resolution of these responses is comparable to or better than current clinical retinal prostheses, the Argus II (reported spatial spreads of approximately 200–4000 μm)^7^ and the Alpha-IMS (reported spatial spreads of ~135–500 μm)^20^. However, the median spatial spread (400 μm) achieved with our prototype device was larger than the resolution (inter-port spacing of 200 μm) of the microports, which supports our earlier conjecture (in reference to Figures 7a–c in the Results section) that glutamate spreads from adjacent microports likely overlapped and thus a larger number of RGCs located in the overlapped region were stimulated by glutamate released from more than one site. Also, the glutamate-evoked RGC pattern responses exhibited robust transient excitation with significantly faster response latencies than visual stimulation and comparatively small time widths suggesting that retinal neurons recovered from exogenous glutamate stimulation on a timescale similar to visual responses. These findings suggest that to achieve more complex injection patterns producing even more focal stimulations, future generation artificial synapse chip devices must comprise a larger array of microports with smaller openings than considered in this study and each microport needs to be activated by an independent, fast-acting, on-chip actuator.

Besides the number and size of ports, other parameters that may influence the efficacy of stimulation in an ultimate chemical synapse chip are: glutamate concentration, pressure or velocity of injections, and depth of injection below the outer surface of the retina. The glutamate concentration (1 mM) and injection pressures (0.69–34 kPa) used in this multisite stimulation study were based on our previous findings of therapeutic ranges of these stimulation parameters using a single port micropipette^12^ delivery system, but the therapeutic ranges of these parameters may be different for *in vivo* stimulation and must be fine-tuned with an implantable synapse chip in retinal degeneration animal models. Furthermore, our extended *in vitro* stimulation experiments did not reveal any glutamate-induced excitotoxic death of retinal neurons because the low concentration (1 mM) glutamate we injected was well below the threshold concentration (3 mM) known to induce excitotoxicity^21^. However, further animal studies employing an implantable chemical synapse chip are required to investigate any potential long-term excitotoxic effects of low concentration glutamate for safe *in vivo* stimulation of the retina.

While subretinal stimulation of the retina with neurotransmitters is a potentially better alternative to electrical stimulation and an artificial synapse chip could be enabled by the state-of-the-art microsystems and microfluidics technologies, a number of challenges will have to be overcome in engineering its topology, materials, design, fabrication, integration with electronics, and *in vivo* testing performed to advance this concept toward its ultimate application for restoration of visual function in patients with retinal degeneration. Some of the major challenges include realizing a device that: is biocompatible, sufficiently thin and flexible to fit in the subretinal space, conformable to the shape of the eye, comprises a high density of independently addressable microports, features on-chip actuators activated by light from the visual field, and has a means to replenish the used-up neurotransmitters over longer periods of operation. Furthermore, although there is evidence suggesting that glutamate stimulation is feasible even in late stages of retinal degeneration^13^, the anatomical and physiological alterations caused by photoreceptor degeneration could present additional unique design challenges to be addressed for realizing an ultimate implantable artificial chemical synapse chip. For instance, the photoreceptor degeneration causes the formation of a glial seal in the subretinal space^22^ that could inhibit the delivery of exogenous neurotransmitters, if injected above the surface of the retina as was done in the current study involving wild-type (normal) retinas. Therefore, an ultimate implantable artificial chemical synapse chip will likely require a high-density array of individually addressable hollow microneedles that can penetrate the glial seal and eject neurotransmitters into the subsurface of the retina near the target INL synapses.

## Conclusions

Neurotransmitter-based stimulation is a promising biomimetic alternative to electrical stimulation for a retinal prosthesis and we have presented the first experimental evidence for feasibility of this stimulation strategy for translating a pixelated pattern of a visual scene into afferent neural signals. We have developed a specialized multiport microfluidic device with independently addressable microports and, by interfacing the device with the retina *ex vivo*, conducted patterned stimulations with exogenous glutamate injections, mimicking the functionality of photoreceptors in the normal retina. When multiple microports were activated in concert to inject glutamate in dot patterns of alphabet characters, clear retinal response patterns that strongly resembled the injection patterns were observed. The spatial resolutions of retinal cells that responded to the patterned glutamate stimulations were comparable to or better than current generation electrical prostheses. As the first prototype artificial mimic of a photoreceptor layer interfaced with *ex vivo* retina, the multiport microfluidic device used in this study represents an early milestone in the development of an artificial chemical synapse chip. These results suggest that an ultimate artificial chemical synapse chip using neurotransmitters could offer high-resolution naturalistic vision to patients affected by photoreceptor degenerative diseases.
